# Vitamin D Status and Health Outcomes in School Children in Northern Ireland: Year One Results from the D-VinCHI Study

**DOI:** 10.3390/nu14040804

**Published:** 2022-02-14

**Authors:** Dominique Ulrike Glatt, Emeir McSorley, L. Kirsty Pourshahidi, Raquel Revuelta Iniesta, Jane McCluskey, Laura Beggan, Mary Slevin, Nigel Gleeson, Diego F. Cobice, Sara Dobbin, Pamela J. Magee

**Affiliations:** 1 Department of Dietetics and Nutrition, Queen Margaret University, Edinburgh EH21 6UU, UK; jmccluskey@qmu.ac.uk (J.M.); ngleeson@qmu.ac.uk (N.G.); 2 Nutrition Innovation Centre for Food and Health (NICHE), Ulster University, Coleraine BT52 1SA, UK; em.mcsorley@ulster.ac.uk (E.M.); k.pourshahidi@ulster.ac.uk (L.K.P.); beggan-l@ulster.ac.uk (L.B.); mm.slevin@ulster.ac.uk (M.S.); 3 Department of Sports and Health Sciences, University of Exeter, Exeter EX1 2LU, UK; 4 Mass Spectrometry Centre, Biomedical Sciences Research Institute (BMSRI), Ulster University, Coleraine BT52 1SA, UK; d.cobice@ulster.ac.uk (D.F.C.); swm.dobbin@ulster.ac.uk (S.D.)

**Keywords:** vitamin D status, vitamin D deficiency, 25(OH)D, healthy school children, Northern Ireland, muscle strength

## Abstract

(1) Background: Vitamin D status has never been investigated in children in Northern Ireland (UK). (2) Methods: Children (4–11 years) (*n* = 47) were recruited from November 2019 to March 2020 onto the cross-sectional study. Anthropometry was assessed. Plasma 25-hydroxyvitamin D (25(OH)D) was analysed. Vitamin D intake, parental knowledge and perceptions, participant habits, physical activity and sedentary behaviour were established via questionnaire. Muscle strength was assessed via isometric grip strength dynamometry and balance via dominant single-leg and tandem stance. Parathyroid hormone, bone turnover markers (OC, CTX and P1NP), glycated haemoglobin and inflammatory markers (CRP, IFN-γ, IL-10, IL-12p70, IL-13, IL-1β, IL-2, IL-4, IL-6, IL-8 and TNF-α) were analysed. (3) Results: Mean (SD) 25(OH)D was 49.17 (17.04) nmol/L (*n* = 47); 44.7% of the children were vitamin D sufficient (25(OH)D >50 nmol/L), 48.9% were insufficient (25–50 nmol/L) and 6.4% were deficient (<25 nmol/L). 25(OH)D was positively correlated with vitamin D intake (µg/day) (*p* = 0.012, r = 0.374), spring/summer outdoor hours (*p* = 0.006, r = 0.402) and dominant grip strength (kg) (*p* = 0.044, r = 0.317). Vitamin D sufficient participants had higher dietary vitamin D intake (µg/day) (*p* = 0.021), supplement intake (µg/day) (*p* = 0.028) and spring/summer outdoor hours (*p* = 0.015). (4) Conclusion: Over half of the children were vitamin D deficient or insufficient. Wintertime supplementation, the consumption of vitamin D rich foods and spring/summer outdoor activities should be encouraged to minimise the risk of vitamin D inadequacy.

## 1. Introduction

Vitamin D is linked to musculoskeletal health as an endocrine regulator of calcium in bone homeostasis [1]. Following the discovery of the vitamin D receptor (VDR) in almost all tissues of the human body [2], the hormonal function of vitamin D has been linked to many other physiological processes and non-skeletal conditions [1,3,4,5,6]. A growing body of evidence suggests that muscle strength and function are positively associated with vitamin D status and may be optimised by vitamin D supplementation [7,8,9]. Vitamin D status in the United Kingdom (UK) is currently clinically defined by circulating 25-hydroxyvitamin D (25(OH)D) concentrations (deficient: <25 nmol/L, insufficient: 25–50 nmol/L, sufficient: >50 nmol/L) and are derived in relation to bone-health outcomes (rickets and osteomalacia) [1,10]. To maintain vitamin D status, the Scientific Advisory Committee on Nutrition (SACN) recommends that all individuals > 1 year of age take a 10 µg (400 IU) vitamin D_3_ supplement daily during the winter months [1], though, this relies on an existing sufficient status. There is disagreement, however, among the clinical and scientific communities (both in the UK and globally) as to what the cut-offs should be [11]. The Endocrine Society recommends higher status cut-offs (deficient: <50 nmol/L, insufficient: 50–75 nmol/L, sufficient: >75 nmol/L) and supplementation to account for non-skeletal health benefits and to maintain 25(OH)D consistently above insufficiency [12]. The most recent National Diet and Nutrition Survey (NDNS) evidence estimates that 19% of children aged 4 to 10 years and 37% aged 11 to 18 years are vitamin D deficient by the end of the winter months and that circulating 25(OH)D is lowest from January to March for all children [13]. The prevalence of vitamin D status in school children is mostly unknown owing to limited research in this area, particularly within the UK. Significant variation in the prevalence of deficiency (4.6–73%) and insufficiency (17–70%) has been reported, likely owing to the heterogenous cohorts studied, and a lack of standardisation in the methods utilised [14,15,16]. These variations are echoed across research from the Republic of Ireland [17,18], the northern hemisphere [19,20,21] and worldwide [22,23], but currently there is no published data on circulating 25(OH)D in school children in Northern Ireland (NI). While the consequences of prolonged vitamin D deficiency in children, i.e., rickets and poor peak bone mass, are well established [24], the full effects of insufficiency are less understood [12].

At present, there is a paucity of evidence investigating the association between vitamin D status and muscle health outcomes in children [7,25,26,27]. There are limited trials, with conflicting results, investigating the effects of vitamin D supplementation on 25(OH)D and muscle strength [27,28,29] and balance [30]. In addition to bone health complications, paediatric hypovitaminosis D has been associated with compromised immunity, arterial stiffness and greater risk of long-term health consequences, including muscle weakness and pain [31,32]. In children who are deficient and insufficient, higher intakes of vitamin D have been associated with reduced risk of infection, type I diabetes mellitus and asthma exacerbation [33,34]. Over the past decade, the role of vitamin D as an innate and adaptive immune modulator has become more apparent, where low circulating concentrations of 25(OH)D have been associated with upper respiratory tract infections and allergic asthma [35,36].

The primary aim of this study was to investigate 25(OH)D concentrations and the vitamin D status of healthy school children aged 4–11 years in NI. A secondary aim of the study was to investigate associations between circulating 25(OH)D and vitamin D status with vitamin D intake, supplementation use, participant vitamin D habits, parental vitamin D knowledge and perceptions, muscle and sensorimotor performance, and bone turnover and inflammatory markers; it is hypothesised that vitamin D inadequacy is associated with compromised outcomes. This paper will discuss the study findings from those children recruited and sampled before the study was postponed due to COVID-19 restrictions.

## 2. Materials and Methods

The D-VinCHI study (*D Vitamin in Children*) is an ongoing multidisciplinary study at Ulster University, investigating vitamin D status in school children and the impact of vitamin D supplementation on 25(OH)D, muscle strength and sensorimotor performance, cognitive function and bone and immune health. Healthy children aged 4–11 years attending school in NI were recruited through university circulations, schools, sports clubs, parent groups, social media (e.g., Facebook) and local advertising. Opt-in recruitment resulted in parents consenting who felt comfortable with their children having a non-clinical blood sample being taken. Children on long-term prescription medication, and/or those who were diagnosed with a long-term or exacerbated health condition or disease were excluded. Children with minor or mild health conditions were included (i.e., occasional asthma). Data collection took place from 1 November 2019 to 17 March 2020 (when the study was suspended due to the COVID-19 pandemic) via a single appointment held at the Human Intervention Studies Unit (HISU) at Ulster University (Coleraine, 55° N). Written parental consent and written participant (child) assent was obtained. Ethical approval was obtained from Ulster University’s Research Ethics Committee (REC/19/0069; clinical trials registration: NCT05018988). This paper reports in accordance with STROBE guidelines [37].

### 2.1. Sample Size

A power calculation (G*Power, Erdfelder, Faul, & Buchner, HHU, Düsseldorf, DE, 1996) estimated a required total sample of 200 participants to investigate 25(OH)D (nmol/L) and vitamin D status (effect size: 0.15; actual power: 0.95). This study presents the results from year one of the DVinCHI study; the sample size in this study (*n* = 47) is comparable with other studies investigating the same outcomes (*n* = 69) [29].

### 2.2. Anthropometry

Participants’ weight (kg) was measured using electronic SECA alpha flat scales (Seca ltd., Hamburg, Germany) and stature (m) was measured using a SECA 213 portable stadiometer (Seca Ltd., Hamburg, Germany) without shoes or outer clothing; body mass index (BMI; kg/m^2^), centiles and BMI z-scores (BMIz) were calculated [38,39]. Waist circumference (WC, cm) was measured without clothing unless children were not comfortable [40,41]. Tricep skinfold thickness (TSF, mm) and mid upper-arm circumference (MUAC, cm) were measured on bare skin [41] and used to calculate upper arm muscle area (UAMA, cm^2^), upper arm fat area (UAFA, cm^2^), arm fat index (AFI, %), arm muscle index (AMI, %) [40] and respective centiles [42]. All measurements were carried out in triplicate and the mean was calculated. Nutritional status was assessed using BMI centiles (very thin: <0.4th, low BMI: <2nd, normally nourished: 2nd–91st, overweight: >91st, obese: >98th and severely obese: >99.6th centile) [38,40,43].

### 2.3. Blood Biomarker Analysis

A 20 mL non-fasted blood (for plasma and serum) sample was obtained by a trained paediatric phlebotomist. Plasma parathyroid hormone (PTH) was analysed using a Cobas 8000 clinical biochemistry analyser (e602 immunoassay module; Roche Diagnostics Ltd., UK) (CVs 1.7%) and plasma glycated haemoglobin (HbA1c) was analysed via HPLC (VARIANT II TURBO HbA_1c_ 2.0; Bio-Rad Laboratories, CA, USA) (CVs 0.8%), both at the Western Trust laboratory in Altnagelvin Hospital. Plasma 25(OH)D was analysed via liquid chromatography-tandem-mass spectrometry (HPLC and MS/MS) (August 2020) using a Reverse Phase (phase A consisted of 50:50 (*v/v*) water: acetonitrile containing 0.1% formic acid as a modifier. Phase B consisted of acetonitrile with 0.1% formic acid) chromatography (Kinetex polar C18 2.6 μm (3.0 × 100 mm) column) and post-chemical derivatisation using turbos pray ionisation in positive ion mode. The multiple reaction monitoring transition for 25(OH)D_3_ was *m/z* 558.3→*m/z* 298.2 and for 25(OH)D2 was *m/z* 570.3→*m/z* 298.2. For the internal standard (25(OH) D3-d6) MRM transition *m/z* 564.4→*m/z* 298.2 was used. Analysis was conducted at the Mass Spectrometry Centre Lab in compliance with the Vitamin D External Quality Assessment Scheme and ICH guidelines for bioanalytical method validation at Ulster University. Vitamin D status was defined by 25(OH)D concentration as follows: deficient: <25 nmol/L, insufficient: 25–50 nmol/L, sufficient: >50 nmol/L [10]. Serum-inflammatory markers (C-reactive protein (CRP) (mg/L), interferon gamma (IFN-γ), tumour necrosis factor alpha (TNF-α) and interleukins (IL): IL-10, IL-12p70, IL-13, IL-1β, IL-2, IL-4, IL-6 and IL-8 (pg/mL)) were assessed via immunoassay (MSD V-Plex Multi-Spot Assay System, K15198D and K15049D, LLC, Merck & Co., Inc., Kenilworth, N.J., U.S.A.) (CVs: 3.96%, 5.93%, 6.71%, 6.96%, 8.01%, 13.79%, 16.24%, 7.63%, 4.01%, 2.89% and 2.51%, respectively) (Ulster University, August 2020) and bone turnover markers via immunoassay (Osteocalcin (OC), C-terminal telopeptide of type 1 collagen (CTX) and Procollagen 1 Intact *N*-terminal propeptide (P1NP), Roche; F. Hoffmann-La Roche Ltd.) (CVs: 0.8%, 1.9% and 1.2%, respectively) (St John’s Hospital, Dublin, Ireland, January 2021).

### 2.4. Questionnaires

Parent-completed questionnaires were used to document participant independent variables: sex, age, school year, post code and skin pigmentation type via an adapted Fitzpatrick scale [44,45]), parental vitamin D knowledge (knowledge score calculation as per O’Conner et al. [46]), habits and perceptions around their child’s vitamin D health and sun exposure [46], their child’s physical activity (Children’s Physical Activity Questionnaire (cPAQ) [47,48]), and their child’s consumption of vitamin D, vitamin D-containing supplements and vitamin D rich food groups (six month retrospective consumption of milk, dairy, cereal products, confectionary, meat, fish and eggs and other fortified foods) via a validated vitamin D specific food frequency questionnaire [49] using the Food Standards Agency’s “The Photographic Atlas of Portion Sizes” portion size estimates (Nelson, Atkinson and Meyer 2002).

### 2.5. Muscle Strength and Balance

Maximal voluntary isometric muscle strength was assessed using age-specific adapted dominant and non-dominant hand-grip strength (kg of force/kg body weight) [50,51,52,53] by dynamometer (TKK 5001 Grip-A analogue dynamometer (64080136); Takei Scientific Instruments Co. Ltd., Niigata, Japan). The test was performed standing with the shoulder and elbow relaxed. Participants were instructed to squeeze as hard as possible for 2–4 s; measurements were repeated three times with a short rest between each test; the mean value of three repeat measures was calculated for each hand. Participant balance was assessed using timed single leg stance (SLS) and tandem stance (TS) according to Condon and Cremin, 2013 [54]. Each test was performed twice, once with the child’s eyes open and once with closed eyes; timing was conducted until the child moved from their stationary position/stepped out of the tandem stance or up to a total of 60 s. All measurements were carried out in triplicate and the mean calculated.

### 2.6. Statistical Analysis

Statistical analyses were conducted using SPSS (Statistical Package for Social Sciences, Version 24.0. SPSS UK Ltd., Chertsey, UK) and figures and tables were created in Microsoft Excel (Microsoft, Redmond, WA, USA). The null hypothesis was rejected at *p* < 0.05. All normally distributed data are presented as mean ± standard deviation (SD), and non-normally distributed data are presented as median (quartile 1 (Q_1_); quartile 3 (Q_3_)). Parametric and non-parametric tests were used where appropriate. The primary outcome measure (vitamin D status) is presented as mean ± SD and percentages. Secondary outcomes were investigated as follows; Spearman’s correlation (*r_s_*) was used to investigate 25(OH)D associations with anthropometry, vitamin D intake, balance, bone turnover and inflammatory outcomes. Pearson’s partial correlation (*r_partial_*) was used to investigate 25(OH)D associations with muscle outcomes corrected for age and anthropometry. Following vitamin D status classification based on 25(OH)D concentrations [10], there were insufficient children in the deficient category (*n* = 3), and therefore they were grouped together with the insufficient status children for statistical comparisons with sufficient children (i.e., non-sufficient (≤50 nmol/L) vs. sufficient (>50 nmo/L)). The Mann–Whitney U (z) test was used to investigate associations between vitamin D sufficiency status and anthropometry, supplementation, vitamin D intake, muscle, balance, bone turnover and inflammatory outcomes. A Chi-square test (Cramer’s V) was used to determine associations between vitamin D sufficiency status and vitamin D habits and perceptions. Single linear regression was run to assess predictability of continuous variable associations with 25(OH)D prior to multiple regression analysis. Multiple linear regression was used to determine predictors of 25(OH)D; non-significant predicators and variables with an inadequate sample size (*n* < 40) were removed from the final model before reporting.

## 3. Results

**Figure 1:**
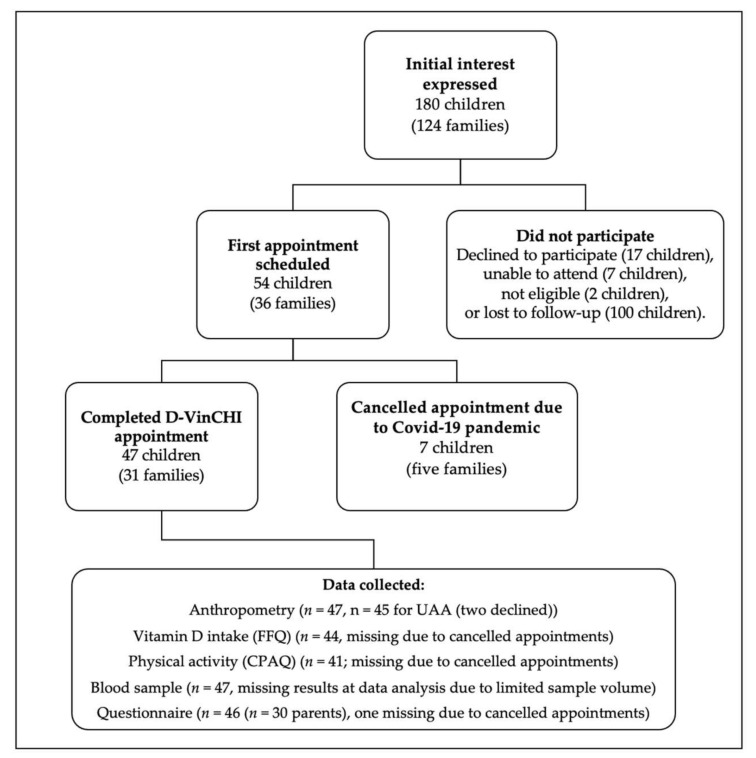
D-VinCHI Recruitment Diagram.

A total of 47 children (60% girls (*n* = 28)) (from 31 families) (Figure 1) with a mean (SD) age of 8 (2) years, completed the study (pre-COVID-19 pandemic). Nutritional status indicated that 2.1% (*n* = 1) of children were underweight (≤2nd percentile), 78.7% (*n* = 37) were a healthy weight (>2nd and <91st), 6.4% (*n* = 3) were overweight (≥91st) and 12.8% (*n* = 6) were obese (≥98th). The children’s anthropometry and upper arm composition data are presented in Table 1. There was no difference between demographic outcomes and anthropometry between males and females. All participants (and parents) identified as white/British or white/other; 77.4% (*n* = 24) of participants’ parents identified as UK nationals, 19.4% (*n* = 6) as Irish nationals (Republic of Ireland) and 3.2% (*n* = 1) as having Romanian nationality. Using the Fitzpatrick skin-type questionnaire [44,45], 61.7% (*n* = 29) of parents descripted their children’s skin type as “pale” (Type II), 34.0% (*n* = 16) as “light brown” (Type III) and 2.1% (*n* = 1) as “moderate brown” (Type IV) skin types. Most parents completed a degree in higher education; 22.6% (*n* = 7) of the parents completed an undergraduate degree, 58.0% (*n* = 18) held a post-graduate degree and 19.4% (*n* = 6) of parents did not have a higher education qualification.

### 3.1. Circulating 25(OH)D and Vitamin D Status

Plasma 25(OH)D concentration (nmol/L) and vitamin D status are presented in Table 2. Mean (±SD) 25(OH)D was 49.17 (±17.04) nmol/L (*n* = 47) and according to NICE guidelines (10), 44.7% of the participants were sufficient (48.9% insufficient, 6.4% deficient) at time of their appointment (November–March 2020). According to the Endocrine Society cut-offs (12) only 8.5% of the participants were sufficient (36.2% insufficient, 55.3% deficient). Some 14.9% (*n* = 7) of the children had a plasma 25(OH)D < 30 nmol/L; 12.5% (*n* = 2) of children aged 4–7 years (*n* = 16) and 16.1% (*n* = 5) of children aged 8–11 years (*n* = 31) had a plasma 25(OH)D < 30 nmol/L. All PTH results were within normal range. Vitamin D sufficient participants had significantly lower median weight (U = 108, z = −2.0, *p* = 0.048), BMI scores (U = 156, z = −2.5, *p* = 0.012), BMIz scores (U = 146, z = −2.7, *p* = 0.007), MUAC (U = 175, z = −2.1, *p* = 0.036), TSF (U = 151, z = −2.3, *p* = 0.022), UAFA (U = 151, z = −2.3, *p* = 0.021), and AFI (U = 160, z = −2.1, *p* = 0.035) compared to those who were non-sufficient; and had higher AMI (U = 345, z = 2.1, *p* = 0.035)) (Table 1).

### 3.2. Supplementation and Vitamin D Intake

Vitamin D intake results are presented in Table 2. Mean (SD) vitamin D intake was 6.4 (5.6) µg/day and mean intake from food alone was 4.4 (2.4) µg/day; sufficient children had a mean intake of 9.0 (7.0) µg/day (4.8 (1.5) µg/day from food sources only) and 4.5 (3.2) (4.1 (2.8) µg/day from food sources) for non-sufficient children. Median total vitamin D intake, vitamin D intake from supplementation and vitamin D intake from cereals were significantly higher in sufficient children compared to non-sufficient children (+1.7 µg/day; U = 335, z = 2.310, *p* = 0.021; +4.2 µg/day; U = 357, z = 2.746, *p* = 0.006; and +1.0 µg/day; U = 337, z = 2.365, *p* = 0.018, respectively). Some 17.0% (*n* = 8) of participants were taking daily vitamin D_3_-containing supplements (vitamin D combination vitamins (*n* = 2) and multivitamins (*n* = 6)); the remaining 83.0% (*n* = 39) were not. Of those not currently reporting supplement use, 41.0% (*n* = 16) would normally take a seasonal supplement (from September/October until March/April), 12.8% (*n* = 5) were not taking any supplement because their parent believed they were getting enough vitamin D, 12.8% (*n* = 5) because the parents stated not being aware of the benefits of supplementation, 10.3% (*n* = 4) because their parents did not know which supplement to use, 7.7% (*n* = 3) for a combination of reasons (enough, unaware of benefits or which supplement to use), 5.1% (*n* = 2) provided other free text responses (“forget” and “undecided”), and 10.3% (*n* = 4) did not provide a reason.

Vitamin D intake was significantly higher in supplement users (17.0% (*n* = 8)) vs. non-supplement users (83.0% (*n* = 39)) (+11.4 µg/day: U = 287, z = 4.352, *p* < 0.001). There was a significant weak positive correlation between 25(OH)D concentration and dietary vitamin D intake (r_s_ = 0.374, *p* = 0.012) and a significant moderate positive correlation between vitamin D intake and the number of vitamin D rich food groups consumed (r_s_ = 0.628, *p* < 0.001). All of the eight participants who were currently taking a supplement and met the daily vitamin D reference nutrient intake (RNI) [1], 87.5% (*n* = 7) had a sufficient vitamin D status (all were taking a supplement containing ≥ 10 µg vitamin D) and 12.5% (*n* = 1) were insufficient (taking a 5 µg vitamin D supplement). The median (IQR) vitamin D intake of participants not taking vitamin D supplementation was 3.7 (4.0) µg and none (*n* = 39) met the UK government’s RNI of 10 µg (400 IU) per day [1]. Those with a vitamin D intake >10 µg/day had a significantly higher median intake of supplements (+8.9 µg/day, U = 306, z = 5.665, *p* < 0.001), cereal (+1.2 µg/day, U = 272, z = 2.866, *p* = 0.004) and fish (+2.1 µg/day, U = 254, z = 2.401, *p* = 0.016) in comparison to those consuming < 10 µg/day. There was a significantly higher prevalence of vitamin D sufficiency (according to NICE [10]) in those taking vitamin D-containing supplements compared to those not taking vitamin D-containing supplements (87.5% vs. 35.9%, Cramer’s V = 0.391, *p* = 0.028), and in those consuming more vs. less than five vitamin D-containing food groups (47.0% vs. 30.0%, Cramer’s V = 12.89, *p* = 0.002). It is noteworthy that the cohort had a mean (SD) milk intake of 460.9 (446.9) mL/day (501.3 (540.1) mL/day and 407.8 (288.0) mL/day for non-sufficient and sufficient children, respectively); however, since none of the milk was fortified, it did not contribute towards vitamin D intake. Using a conservative 1.5 µg/100 g fortification concentration, milk fortification would have resulted in an additional 6.9 µg/day of vitamin D (7.5 µg/day and 6.0 µg/day for non-sufficient and sufficient children, respectively) [55].

### 3.3. Participant Vitamin D Habits, and Parental Vitamin D Knowledge and Perceptions

Circulating 25(OH)D was positively correlated with median hours per week spent outdoors during the spring and summer months (moderate correlation, *r_s_* = 0.402, *p* = 0.006), and negatively correlated with median screen time (h/week) (weak correlation, *r_s_* = −0.334, *p* = 0.023) and sedentary time (h/week) (moderate correlation, *r_s_* = −0.486, *p* = 0.001) (Table 2). There was a significant difference between those with sufficient and non-sufficient vitamin D status in median outdoor spring and summer time (+7 h/week; U = 3.8, z = 2.4, *p* = 0.015), median screen time (−3.5 h/week; U = 3.8, z = 2.4, *p* = 0.015) and median sedentary time (−20.2 h/week; U = 3.8, z = 2.4, *p* = 0.015), respectively (Table 2). The median parental vitamin D knowledge score was 39.2 (79.7) %; median scores were significantly higher in parents with a nutrition qualification compared to those without a nutrition qualification (+41.0%, U = 10, z = 3.88, *p* < 0.001). After controlling for nutrition qualifications, however, there was no significant correlation between knowledge score and 25(OH)D. Parents who assumed that food sources were sufficient did not administer supplements (currently or seasonally) to their children. When asked about vitamin D fortification, 76.6% (*n* = 23) of parents did not believe there was “any harm in eating or drinking fortified food” (20.0% replied unsure (*n* = 6); 3.3% replied yes (*n* = 1), clarification: “due to fear of overdose”); and 90.0% (*n* = 27) of parents were willing to buy vitamin D fortified foods for their children ((6.7% were unsure (*n* = 2), and 3.3% was unwilling (*n* = 1)). No other significant associations were observed.

### 3.4. Muscle and Sensorimotor Performance

Muscle and sensorimotor outcomes are presented in Table 2. There was a significant, albeit weak, positive correlation between 25(OH)D and dominant grip strength (kg) (*r_partial_* = 0.317, *p* = 0.044) after controlling for age, WC, BMI, UAMA and UAFA. No further correlations with balance or between vitamin D status categorisation were detected.

### 3.5. Bone Turnover Markers, HbA1c and Inflammatory Markers

Values for bone turnover markers (*n* = 47), HbA1c (*n* = 37) and inflammatory marker (*n* = 46) results are shown in Table 3. There was a moderate negative correlation between plasma-25(OH)D and serum IL-13 (*r_s_* = −0.444, *p* = 0.002), and a statistically significant difference in median serum IL-13 concentration between those who were sufficient (0.1 (0.0, 0.2) pg/mL) and non-sufficient (0.5 (0.3, 0.8) pg/mL) (U = 115, z = −3.4, *p* < 0.001). No further associations were detected.

### 3.6. Predictors of Plasma 25(OH)D

Single linear regression identified significant associations between 25(OH)D and PTH (*p* = 0.02), II-13 (*p* = 0.009), vitamin D intake (*p* = 0.028), number of vitamin D-containing food groups (*p* = 0.045), weekly screen time (*p* = 0.022), weekly sedentary hours (*p* = 0.028) and spring/summer sun exposure (*p* = 0.002). Multiple regression found that total sedentary hours (β = −0.40, *p* = 0.007) and circulating IL-13 (β = −0.41, *p* = 0.006) statistically predict 25(OHD) concentrations in this cohort, F (4, 38) = 7.4, *p* = 0.002, adj. R^2^ = 0.281 (Table 4). No further significant predictors were detected.

## 4. Discussion

These novel results from the D-VinCHI study are the first summary of wintertime vitamin D status prevalence in school children in NI, reporting that more than half of the group had an insufficient or deficient vitamin D status (25(OH)D < 50 nmol/L). This result demonstrates that vitamin D inadequacy remains a risk in the UK owing to its northerly latitude limiting the opportunity for endogenous synthesis, together with low intakes of vitamin D [13,23], despite attempts at mitigating these risks via voluntary fortification [55,56] and supplementation campaigns [57]. The prevalence of deficiency is lower in the D-VinCHI cohort (6.4%) compared to that of the UK population (19%) [13]. A separately published NDNS sub-cohort study (*n* = 167) indicating a 10% deficiency (<25 nmol/L) and 48% insufficiency (<50 nmol/L) for 4–10 year olds [58] presented more comparable findings of the current study. This is further supported by an Avon Longitudinal Study cohort (*n* = 5800, 7.3–13.6 years), which reported that 29% of the participants had a 25(OH)D concentration < 50 nmol/L [16]. Two Irish studies observed similar levels of vitamin D inadequacy; one with a toddler cohort (*n* = 294, average (SD) 25(OH)D concentration 54.5 (19.9) nmol/L, 45.2% < 50 nmol/L) [17] and one with a children and adolescent cohort (*n* = 252, average (SD) 25(OH)D concentration 51 [25] nmol/L, 55% < 50 nmol/L) [56]. Vitamin D inadequacy remains a global concern, particularly in the northern hemisphere, where incidence rates of vitamin D associated chronic musculoskeletal and health issues are higher [59,60]. Furthermore, the current UK vitamin D status definition does not account for non-skeletal health outcomes and pathologies associated with higher cut-offs for vitamin D (e.g., cancer risk, CVD, muscle strength, and immunity), suggesting that an even greater proportion of the population may have sub-optimal 25(OH)D [12,23].

In the current study, vitamin D sufficient children had higher intakes of total vitamin D, vitamin D from supplements and vitamin D from cereal products. Even when accounting for supplement use, median daily vitamin D intake (4.2 µg) remained below the UK recommended lower reference nutrient intake (LRNI) of 10 µg/day, supporting previous reports in the UK and across Europe [13,18,61]. Higher 25(OH)D, a sufficient vitamin D status and the consumption of >10 µg vitamin D/day were all associated with vitamin D supplement intake. These results are comparable to similar prevalence studies [15,16,17,56,62], which all observed a positive association between vitamin D intake from supplements and circulating 25(OH)D and/or an increase in the percentage of the population meeting the vitamin D LRNI. Supplementation is a well-documented method of correcting and preventing vitamin D deficiency [1,63]; however, the adherence to supplementation is a leading factor in long-term improvement of vitamin D status and is currently insufficient in the UK [23,64]. Adherence may be improved in children by encourage parents to take a seasonal vitamin D supplement, if both parents and children are equally responsible for administering or if supported with health surveillance (regular checks by a health care professional) [65]. In the current study, after supplementation, fortified foods (cereals and fat spreads) significantly contributed towards an intake greater than 10 µg/day, and the top three consumed food groups were fortified cereals, fish and fortified spreads. In the UK, cereal and cereal products contribute to 13–20% of the population’s vitamin D intake [13,58]; research shows that multiple sources of fortification and regular consumption of these foods can contribute towards higher 25(OH)D [58,66,67] and may contribute towards deficiency prevention [68]. There is evidence to suggest that mandatory fortification, instead of voluntary, may be necessary to have a population wide impact [69,70]. Food fortification improves 25(OH)D [71], reduces the necessity of supplement adherence and may reach a wider population and increased impact [72,73]. Successful national fortification schemes have increased vitamin D intake by 11 μg/day (440 IU/day) and increased circulating 25(OH)D by 19.4 nmol/L [72]; in the current study, a 1.5 µg/100 g milk fortification would have resulted in the mean vitamin D intake meeting the UK LRNI [55]. The Finish liquid dairy and fat spread fortification initiative increased vitamin D intake by 12–15 μg/day over a decade [73]. In the current study, consuming > five vitamin D rich food groups was associated with higher daily vitamin D intake and 25(OH)D. Consuming fewer food groups, however, could be a risk factor for children with picky-eating behaviour (not assessed in this study), including children diagnosed with Autism Spectrum Disorder [74,75]. Picky or restrictive eating is known to affect nutrient intake, which may result in compromised growth and health outcomes (i.e., hypovitaminosis D) [75].

We observed that vitamin D sufficient children had lower circulating cytokine IL-13. Circulating 25(OH)D tends to be negatively associated with proinflammatory cytokines (i.e., IL-2, IL-9, IL-13, IL-22, interferon-γ and tumour necrosis factor α), and positively associated with anti-inflammatory cytokines (i.e., IL-3, IL-4, IL-5 and IL-10) [35]; however, research in healthy children is limited. IL-13 is involved in immune defense and allergic inflammatory responses, including asthma, as part of the Th2 pathway, and has a role in converting 25(OH)D_3_ to its active metabolite 1,25(OH)_2_D_3_ in bronchial epithelial cells [76]. Vitamin D has been shown to downregulate allergen induced expression of IL-13 [77], suggesting an immunoregulatory role and reduction of airway inflammation and hyperreactivity, and vitamin D deficiency has been associated with asthmatic symptoms, including wheezing and respiratory infections [78]. Thus, those with hypovitaminosis D may be more susceptible to respiratory allergic response or asthma. As this was not captured in this study, future studies may benefit by querying participant allergy history.

There was a weak significant association found between 25(OH)D and dominant grip strength after controlling for age and anthropometry, suggesting a role for vitamin D in muscle strength within this group. Few studies have investigated circulating 25(OH)D and physical performance in healthy children, using a wide range of methods: isometric strength measures (i.e., leg and handgrip strength), vertical jump height, sprint tests, agility tests, balance, exercise time, physical activity level and even “time outdoors” [7,25,26,27]. The majority of studies have shown a positive correlation between 25(OH)D and physical performance (mainly muscle strength) [7,25,28,29,79,80,81]; however, others have reported no association [27,29]. While the current SACN report on vitamin D [1] included only one study investigating vitamin D status and muscle health in children [29], more recent research supports a positive relationship between 25(OH)D and muscle strength [81]. Similarly, in a cohort of 5-year-old children, 25(OH)D was associated with hand grip strength in girls, once controlled for anthropometry and body composition [7]. Balance establishes an individual’s musculoskeletal functional capability and is often used clinically to assess physical recovery after injury [81]. The current study observed no associations between 25(OH)D or vitamin D status and balance measures. Balance may be affected by age as others have reported that balance may only mature after the age of 10 years (*n* = 94) [82]; however, a greater sample size is needed to fully investigate this. Increasingly, associations between 25(OH)D and muscle health outcomes tend to become more evident when investigating change in 25(OH)D instead of just prevalence [28,29,30].

Vitamin D sufficient children had significantly higher total hours outside during the spring and summer, and less total sedentary hours and screen time. Moreover, weekly sedentary hours were a negative predictor of 25(OH)D. These findings are supported by an Avon longitudinal study sub-cohort, which found time spent outside correlated with 25(OH)D_3_ [16]. Furthermore, a Belgian study found that outside playing hours correlated with 25(OH)D (*n* = 357, r = 0.140, *p* < 0.001) and predicted 25(OH)D [83]; in a Danish study (*n* = 130), 25(OH)D was higher in active children vs. less active children (+5.6 (95% CI 1.1, 10.0) nmol/L) [84]. These results present evidence for possible lifestyle predictors of 25(OH)D (e.g., outside time), which could serve as simple status indicators instead of needing to assess circulating 25(OH)D. The clinical or public health application of asking a simple question (e.g., “Are you sedentary for most of the day?”) or promotion of outdoor programs (e.g., for children who do not have access to a safe outdoor space) could help prevent deficiency, save money, and avoid invasive procedures. Further research is needed to establish the efficacy and applicability of these measures.

### Limitations and Future Research

Unfortunately, owing to COVID-19 and the suspension of the study, the full sample size was not achieved, and missing data was more prevalent owing to a lack of possible follow-up; therefore, generalisability may be reduced and statistical inference is limited to this cohort. Significant correlations were observed; however, these need to be confirmed in the larger cohort, though their inclusion in this study is still important as they potentially evidence associations worth investigating in the future. Additionally, this study provides novel data on the prevalence of pre-COVID-19 pandemic vitamin D inadequacy in school children from NI, particularly given the associations between vitamin D hypovitaminosis and immune health [31,32].

## 5. Conclusions

To our knowledge, this is the first study to investigate the vitamin D status in a well-characterised cohort of school children in NI. Our findings indicate that 55.3% of children are insufficient or deficient during winter and suggest that low circulating concentrations of 25(OH)D may adversely affect muscle strength. Encouraging supplementation, consumption of vitamin D rich foods and the promotion of outdoor activities should be considered to improve vitamin D status and minimise the risk of deficiency. Further research investigating vitamin D status and the efficacy of preventing insufficiency and deficiency during the winter months via supplementation or consumption of vitamin D rich foods in school children in NI is warranted.

## Figures and Tables

**Table 1:** Participant Demographics and Anthropometry †.

	*n* = 47	Non-Sufficient ¶ (*n* = 26, 55.3%)	Sufficient ¶ (*n* = 21, 44.7%)
Decimal age (years)	8.0 ± 3.0	9.0 ± 2.0	8.1 ± 2.4)
Sex at birth (female (*n* (%)))	28 ± 59.6	15 ± 57.7	13 ± 61.9
Height (cm)	133.7 ± 14.5	135.8 ± 16.3	132.1 ± 11.2
Weight (kg)	28.4 (24.4; 40.6)	27.7 (24.8; 48.3)	28.8 (20.9; 33.8) *
BMI (kg/m^2^)	16.6 (15.4; 19.7)	16.9 (16.2; 20.5)	17.0 (15.0; 18.3) *
BMIz (kg/m^2^)	0.2 (−0.4; 1.2)	0.6 (−0.1; 1.5)	0.5 (−0.7; 0.9) **
Waist circumference (cm)	56.3 (53.2; 64.2)	56.7 (54.5; 66.5)	55.1 (50.6; 62.5)
MUAC (cm)	19.2 (18.3; 25.1)	19.6 (18.9; 25.3)	19.2 (17.3; 22.1) *
TSF (mm) ^‡^	10.8 (8.5; 17.7)	11.1 (9.1; 16.4)	10.0 (7.4; 17.7) *
UAMA (cm^2^)	21.0 (19.1; 27.9)	21.6 (19.2; 30.0)	20.2 (18.1,; 25.3)
UAFA (cm^2^) ^‡^	8.8 (7.2; 19.3)	10.7 (7.5; 15.7)	8.8 (6.2, 19.0) *
AMI (%) ^‡^	68.2 ± 8.7	67.8 ± 9.3	68.2 ± 8.5 *
AFI (%) ^‡^	30.1 (26.0; 40.1)	31.5 (27.0; 41.1)	29.4 (24.8; 39.0) *

Abbreviations: SD, standard deviation; Q, quartile; BMI, body mass index; BMIz, BMI z-score; MUAC, mid upper arm circumference; TSF, tricep skinfold thickness; UAMA, upper arm muscle area; UAFA, upper arm fat area; AMI, arm muscle index; AFI, arm fat index. † Mean ± SD or median (Q1; Q3), all such values. All anthropometry was measured in triplicate, mean was obtained. ¶ Circulating 25(OH)D concentration unit conversion: 2.5 nmol/L = 1 ng/mL. Vitamin D status cut-offs; sufficiency ≥50 nmol/L, nonsufficient <50 nmol/L (includes both insufficient (50–25 nmol/L) and deficient (<25 nmol/L) classifications). ‡ Missing data: *n* = 45 for all such measurements (missing: 2 females, 0 males) as two children were unwilling to have the TSF measurement taken. Significant difference between vitamin D sufficiency vs. non-sufficiency; Mann–Whitney U test: * *p* < 0.05, ** *p* < 0.01.

**Table 2:** Circulating Plasma 25-hydroxyvitamin D Concentrations, Vitamin D Status, Vitamin D Intake, Muscle and Sensorimotor Performance, Activity and Sedentary Outcomes †.

	*n* = 47 ^‡^	Non-Sufficient ¶ (*n* = 26, 55.3%)	Sufficient ¶ (*n* = 21, 44.7%)
25(OH)D (nmol/L)	49.2 ± 17.0	34.6 ± 8.8	65.26 ± 9.8
PTH (ng/mL) (optimal range: 15–65 ng/mL)	31.3 ± 10.9	34.2 ± 10.9	28.0 ± 9.8
**Dietary vitamin D intake ^‡^** ^§^			
Total Vitamin D Intake (µg/day)	4.2 (2.8; 7.6)	3.5 (1.8; 6.9)	5.2 (3.7,; 14.8) *
from Supplement (µg/day)	1.9 ± 4.5	nil	4.2 ± 6.0 *
from Food (µg/day)	4.1 (2.8; 6.1)	3.4 (1.8; 6.1)	4.7 (3.7; 6.2)
Number of vitamin D rich food groups	7.0 (6.0; 8.0)	6.0 (5.0; 8.0)	7.0 (7.0; 8.0)
**Top-five contributing food groups**:			
Cereals (fortified) (µg/day)	1.3 (0.3; 2.0)	0.6 (0.2; 1.9)	1.6 (0.3; 2.3) *
Fish (µg/day)	0.6 (0.0; 1.8)	0.1 (0.0; 0.8)	1.5 (0.1; 1.9)
Fat spreads (fortified) (µg/day)	0.5 (0.2; 0.9)	0.3 (0.1; 1.0)	0.6 (0.3; 0.8)
Meat (µg/day)	0.3 (0.2; 0.9)	0.3 (0.1; 1.4)	0.3 (0.2; 0.8)
Eggs (µg/day)	0.3 (0.2; 0.5)	0.3 (0.2; 0.5)	0.3 (0.2; 0.4)
**Muscle and Sensorimotor Performance**			
Dominant grip strength (kg)	12.3 ± 5.0	12.4 ± 5.4	12.0 ± 5.3
Non-dominant grip strength (kg)	11.3 ± 4.7	11.0 ± 5.0	11.1 ± 5.1
Balance: SLS eyes-open (s)	25.7 (11.4; 45.3)	28.1 (13.7; 46.1)	27.2 (11.9; 50.2)
Balance: SLS eyes-closed (s)	4.7 (3.2; 6.9)	4.5 (3.2; 6.5)	4.5 (3.4; 7.1)
Balance: TS eyes-open (s)	41.1 (28.4; 52.8)	44.6 (30.9; 60.0)	39.2 (35.1; 50.5)
Balance: TS eyes-closed (s)	9.7 (5.4; 17.7)	10.9 (5.6; 15.6)	9.2 (5.3; 22.2)
**Activity and Sedentary Outcomes**			
Active time (h/week)	10.7 ± 5.8	8.8 ± 7.1	10.6 ± 6.4
Number of activities	2.8 ± 1.8	2.7 ± 2.1	2.9 ± 1.3
Spring/summer outside time (h/week)	22.2 ± 12.3	15.3 ± 11.0	27.3 ± 13.3 *
Sedentary time (h/week)	37.5 (25.1; 46.5)	43.5 (36.8; 54.1)	35.8 (21.4; 46.3) *
Screen time (h/week)	10.5 (6.6; 17.6)	12.3 (6.3; 21.0)	10.5 (3.8; 17.5) *

Abbreviations: SD, standard deviation; Q, quartile; 25(OH)D, plasma-25-hydroxy vitamin D; PTH, parathyroid hormone; SLS, single leg stance; TS, tandem stance; h, hours. Active time, sedentary time and number activities were derived from the Children’s Physical Activity Questionnaire. Screen time (TV, computer, tablet, mobile phone, car viewing devices, cinema, video or computer games, hand-held game devices (e.g., Nintendo)) estimated from parent-completed questionnaire; screen time was used for entertainment and schoolwork. † Mean ± SD or median (Q1; Q3), all such values. All muscle and sensorimotor performance were measured in triplicate, mean was obtained. ¶ Circulating 25(OH)D concentration unit conversion: 2.5 nmol/L = 1 ng/mL. Vitamin D status cut-offs; sufficiency ≥ 50 nmol/L, nonsufficient < 50 nmol/L (includes both insufficient (50–25 nmol/L) and deficient (<25 nmol/L) classifications). ‡ Missing data: *n* = 43 (missing: 4 participants) for PTH data due to COVID-19 March lockdown and repeats were not possible as a result; *n* = 44 (missing: 3 participants) for vitamin D intake due to incomplete questionnaire and parents lost to follow-up; *n* = 43 and *n* = 41 for eyes open and eyes closed measurements respectively, due to children refusing measurement (*n* = 2) and time limitations (*n* = 4); *n* = 41 cPAQ results (missing: 6 participants) and *n* = 46 for questionnaire results (spring/summer outside time and screen time) (missing: 1 participant) lost to follow-up. § Daily vitamin D intake derived from a validated six-month retrospective vitamin D specific FFQ [49]. Vitamin D unit conversion: 1 µg = 40 IU. Significant difference between vitamin D sufficiency vs. non-sufficiency; Mann–Whitney U test: * *p* < 0.05.

**Table 3:** Biomarkers of bone turnover, blood glucose and inflammation †.

	*n* = 47 ^‡^	Non-Sufficient ¶ (*n* = 26, 55.3%)	Sufficient ¶ (*n* = 21, 44.7%)
Osteocalcin (µg/L)	97.7 (78.9; 118.0)	100.1 (86.8; 116.7)	89.8 (78.1; 117.2)
P1NP (µg/L)	513.9 (437.7; 778.1)	504.4 (441.2; 908.0)	521.3 (418.8; 706.5)
CTX (pg/mL)	1.2 (1.0; 1.7)	1.3 (1.1; 1.7)	1.2 (1.0; 1.3)
Plasma HbA1c (mmol/mol)	32.1 ± 3.0	33.0 ± 1.7	31.5 ± 2.4
CRP (mg/L)	0.3 ± 0.7	0.4 ± 0.1	0.8 ± 1.4
IFN-γ (pg/mL)	5.2 (3.3; 11.6)	5.1 (3.3; 14.6)	6.4 (4.3; 14.0)
IL-10 (pg/mL)	0.4 (0.2; 0.6)	0.3 (0.2; 0.6)	0.4 (0.4; 0.6)
IL-12p70 (pg/mL)	0.1 (0.0; 0.2)	0.1 (0.0; 0.2)	0.1 (0.0; 0.2)
IL-13 (pg/mL)	0.3 (0.1; 0.6)	0.5 (0.3; 0.8)	0.1 (0.0; 0.2) ***
IL-1β (pg/mL)	0.1 (0.0; 0.2)	0.1 (0.0; 0.2)	0.0 (0.0; 0.1)
IL-2 (pg/mL)	0.4 (0.2; 0.4)	0.3 (0.2; 0.5)	0.2 (0.2; 0.3)
IL-4 (pg/mL)	0.01 (0.00; 0.02)	0.01 (0.00; 0.02)	0.01 (0.01; 0.02)
IL-6 (pg/mL)	0.5 (0.3; 0.6)	0.5 (0.3; 0.6)	0.4 (0.2; 0.6)
IL-8 (pg/mL)	9.4 (7.3; 12.4)	8.6 (7.0; 11.6)	8.8 (7.4; 9.9)
TNF-α (pg/mL)	3.2 ± 0.8	2.4 ± 0.5	3.1 ± 0.9

Abbreviations: SD, standard deviation; Q, quartile; HbA1c, glycated haemoglobin; P1NP, procollagen 1 intact *N*-terminal propeptide; CTX, C-terminal telopeptide of type 1 collagen; CRP, C-reactive protein; IFN-γ, interferon gamma; IL-10, -12p70, -13, -1β, -2, -4, -6, -8, interleukin 10, 12p70, -13, -1beta, -2, -4, -6, -8 and IL-12p70 (respectively); TNF-α, tumour necrosis factor alpha. Reference ranges: plasma-HbA1c: <48 nmol/mol; and CRP: <5 mg/L; IFN-γ < 4.2, IL-10 < 2.8, IL-12p70 < 1.9, IL-13 < 2.3, IL-1β < 6.7, IL-2 < 2.1, IL-4 < 2.2, IL-6 < 16.4, IL-8 < 9.4 and TNF-α < 29.4. All biochemistry markers were analysed from serum aside from HbA1c (plasma). † Mean ± SD or median (Q1; Q3), all such values. All anthropometry was measured in triplicate, mean was obtained. ¶ Circulating 25(OH)D concentration unit conversion: 2.5 nmol/L = 1 ng/mL. Vitamin D status according to NICE Guidelines (2016) [10] status cut-offs; sufficiency ≥ 50 nmol/L, nonsufficient < 50 nmol/L (includes both insufficient (50–25 nmol/L) and deficient (<25 nmol/L) classifications). ^‡^ Missing: n = 37 (missing: 10 participants) HbA1c results due to insufficient blood sample for lab analysis. *n* = 46 for all inflammatory markers as one outlier removed as they were >3×SD. Significant difference between vitamin D sufficiency vs. non-sufficiency; Mann–Whitney U test: *** *p* < 0.001.

**Table 4:** Factors predicting plasma 25-hydroxyvitamin D concentration determined using multiple regression analysis.

	B	95% CI for B		*p*-Value	R^2^
		LL	UL		
Plasma-25(OH)D (nmol/L) (*n* = 40)				0.002 **	0.281
IL-13 (pg/mL)	−14.835	−25.068	−4.603	0.006 **	
Sedentary time (min/week) *(cPAQ)*	−0.005	−0.008	−0.001	0.007 **	

Abbreviations: 25(OH)D, plasma-25-hydroxy vitamin D; IL-13, interleukin 13; B, unstandardised regression coefficient; CI, confidence interval; LL, lower limit; UL, upper limit; R^2^, coefficient of determination. Significance: ** *p* < 0.01.
